# What’s the Use

**Published:** 1911-03

**Authors:** 


### "What's The Use."

With the approach of the time for another legislative session to convene, the following extracts from editorials of prominent medical journals make interesting reading and should help us to get in proper frame of mind for the work before us:

"It is discouraging to see how tenaciously the public clings to prejudices, especially when the medical profession consistently aim to remove these prejudices. Perhaps people receive as much protection as they deserve, and ignorant legislators, reflecting the narrow views of their constituents, enact only such laws as will dispel a little darkness, for fear that the full glare of enlightened liberty may prove fatal. Whenever the medical profession urges measures of health reform, lawmakers bristle up at once, and antagonize them, forgetting that the prevention of disease is in direct opposition to the profession's material interests. The very humanitarianism of the demands for health reform cause physicians to be misjudged, since no other profession or calling is so insistent in the matter of working against its own material interests. Physicians at times almost feel that the public should be permitted to learn common sense in the bitter school of adversity, without any effort of true lovers of their kind to keep them from the results of their folly. And yet the medical profession would be untrue to its trust if it failed in season and out of season to teach people the rules of health and longevity."—*Cincinnati Lancet-Clinic.*

"What's the use?" Why should we, the honest, hardworking medical men of the country, go on fighting so strenuously to prevent disease and cut down its ravages? Try as we may, our motives are misconstrued, our most earnest efforts are looked upon with suspicion, and our results are belittled and besmirched.

Why should the medical profession keep on fighting to protect the people from their own follies, when success means a decrease of disease and an inevitable decline in professional incomes? Why do we go on striving to perfect health laws when such laws rob us of our means of livelihood?

The answer is not difficult to determine. Every earnest physician merges himself in his work as do men in few other callings. The practice of medicine is a humane profession. All unconsciously the spirit of unselfishness creeps into a true doctor's life, and sooner or later he finds himself living not for himself but for humanity. There are plenty who will scoff at this and ridicule the idea. But every doctor who reads this knows that there is an exaltation felt by those who minister unto the sick and suffering that makes the active practice of medicine the most gratifying, most satisfactory calling on earth. It is good to be a doctor of medicine, to feel the responsibilities one is forced to assume, and then to realize the trust and confidence that patients give to us in their hours of greatest distress and anguish. It is these very things that make the practice of medicine ennobling in many ways that only those engaged in its pursuits can understand. If the laity could only grasp and analyze the psychic effects of the sense of personal responsibility that becomes a real force in every honest doctor's life, there would be a great deal more sincere respect entertained by those who are now all too prone to criticise and condemn.

And so in spite of being misunderstood, of being slandered and falsely accused of the basest motives, every earnest physician will go on fighting for the right as he sees it. Let the jackals howl and the snakes hiss, our duty is plain and whether we win or not we can never be robbed or cheated of the benefits that invariably accrue from doing our best. The true apotheosis of medical practice has ever come from its least appreciated and sometimes most actively combated efforts in behalf of indifferent or unwilling humanity.—*American Medicine.*

---

The Rockefeller Institute for Medical Research announced on February 12th that the effectiveness of anti-meningitis serum had been generally accepted by medical authorities throughout the world and that the new remedy had taken its place with vaccine and diphtheria antitoxin "as an improved agency for the protection of public health."

The occasion for this announcement was the notice given by the institute that hereafter it would discontinue free distribution of the serum which has been carried on since its discovery and devote the funds to other lines of investigation.

The New York City Board of Health has undertaken the production of the new serum, and for a short time will supply urgent requests from outside the State.

